# Online-delivered ‘Teaching Recovery Techniques’ for young people with PTSD symptoms who have experienced forced migration: a pilot study

**DOI:** 10.1016/j.invent.2026.100918

**Published:** 2026-02-13

**Authors:** Georgina Warner, Sandra Gupta Löfving, Emma Geijer-Simpson

**Affiliations:** a Child Health and Parenting (CHAP), Department of Public Health and Caring Sciences, Uppsala University, Uppsala, Sweden

**Keywords:** Online delivery, Trauma-Focused Intervention, Post-Traumatic Stress Disorder, Forced Displacement, Teaching Recovery Techniques, Young People

## Abstract

**Introduction:**

Although there is a high need for mental health support among individuals with experience of forced migration, there are barriers to accessing in-person interventions. Online delivery offers an alternative. This study aimed to examine the feasibility and acceptability of online-delivered Teaching Recovery Techniques (TRT) for young people who had experienced forced migration and reported symptoms of PTSD.

**Methods:**

The study used an open, single-arm trial design with a mixed-methods approach. Participants (*n* = 16; 62.5% male; 17–23 years) were recruited via an upper secondary school in Sweden. Fidelity checklists were used to capture adherence to the manual, psychological symptom and life satisfaction questionnaires were administered (*n* = 16), and a focus group discussion (*n* = 3) and interview (n = 1) explored participants' perspectives.

**Results:**

High fidelity was observed, with all components delivered except for elements of the final session. Technical challenges were noted, including limited platform functionality for private communication and unstable internet connectivity, and privacy concerns were raised where participants lacked private spaces. The format was adapted, including merging groups, delivering sessions twice weekly rather than weekly, and reducing session duration from 90 to 60 min. Of 16 participants, 9 completed post-intervention measures; descriptive data suggested completers were less likely to be female and had higher symptom scores. Qualitative data indicated symptom improvements and emphasised facilitators' relational qualities, but benefits were described as transient and insufficient to address ongoing stressors, with participants preferring in-person delivery.

**Conclusion:**

Online TRT needs enhanced technical support, privacy safeguards, and closure procedures; future trials should test efficacy and long-term outcomes.

## Highlights

• Online TRT was delivered with high fidelity, except elements of the final session.
• Technical and privacy constraints limited feasibility of online group delivery.
• Session frequency and duration were modified to maintain participation.
• Acceptability was mixed; benefits were transient and in-person delivery preferred.

## 1 Introduction

Post-traumatic stress disorder (PTSD) is a psychiatric condition characterised by persistent re-experiencing, avoidance, negative alterations in cognition and mood, and heightened arousal following exposure to a traumatic event (American Psychiatric Association, 2013). Lifetime prevalence of PTSD is estimated to be 4.7% (McLaughlin et al., 2013); however, it is substantially higher among refugee children and young people (CYP), as they are exposed to traumatic events to a larger extent (Betancourt et al., 2017). This exposure may be in their country of origin (war exposure, violence, loss of family members), leading them to seek refuge elsewhere as well as during migration (dangerous journeys, exploitation, detention) (Abdi et al., 2023). These trauma experiences may be exacerbated by resettlement stress as refugee CYP deal with discrimination, social isolation, acculturation and uncertain legal status during a developmentally critical time (Abdi et al., 2023; Fazel and Betancourt, 2018). Studies indicate rates of PTSD between 19% and 54% among refugee CYP in high-income countries (Bronstein and Montgomery, 2011). Different assessment methods influence these rates; clinical interviews reveal that about one in four refugee CYP meets PTSD criteria (Blackmore et al., 2020). In Sweden, accompanied CYP have threefold the risk of PTSD compared to Swedish-born peers (Mittendorfer Rutz et al., 2020), while unaccompanied refugee CYP face an 8-fold higher risk (Björkenstam et al., 2020), highlighting the protective role of caregivers.

PTSD care for CYP consists of three levels: self-help, non-specialist care, and specialist care. Teaching Recovery Techniques (TRT) (Smith et al., 1999; Yule et al., 2013) is an example of non-specialist care. It is a 5-week, evidence-based group intervention that focuses on trauma symptom management techniques for CYP. The intervention approach aligns closely with trauma-focused cognitive behavioural therapy (TF-CBT). Participants learn to recognise the effects of trauma, along with coping skills to manage flashbacks and intrusive sensations and techniques for stabilisation and relaxation to address hyperarousal and gradually desensitise avoidance behaviours. TRT has been evaluated with CYP in several international settings including Australia (Ooi et al., 2016), Brazil (Barron et al., 2021), China (Chen et al., 2014), Norway (Oppedal et al., 2019), Pakistan (Abbas et al., 2024), Palestine (Barron et al., 2016; Barron et al., 2013; Qouta et al., 2012), Sweden (Sarkadi et al., 2018), Thailand (Pityaratstian et al., 2015), the UK (Barron et al., 2017), and Ukraine (Yavna et al., 2024) and an overall positive impact on symptoms of PTSD and depression has been reported (Hedges' g = −0.53 [−0.97, −0.08]) (Davis et al., 2023).

To meet the need for online delivery of TRT during the COVID-19 pandemic, Pérez-Aronsson et al. (2022) conducted a participatory adaptation and usability test with refugee youth in Sweden. The project involved a qualitative needs assessment with TRT group leaders and youth, participatory workshops with refugee youth, consultation with refugee parents and trauma professionals, and usability testing materials with group leaders and refugee youth. Key findings highlighted the importance of clear guidance on safety protocols, strategies for fostering online group cohesion, and technical accessibility. Adaptations included revising the manual language and developing visual and audio resources. While the online format was generally found to be acceptable and the resources usable, the study underscored the value of detailed planning and stakeholder involvement when transitioning interventions to virtual platforms. Since then, there has been some delivery of TRT via remote modalities (Yavna et al., 2024); however, this has occurred as an ad hoc response to wartime logistical constraints, and its feasibility and acceptability have not yet been systematically evaluated.

As teleconferencing becomes more common for delivering psychological interventions, especially in contexts where in-person access is limited, research on the effectiveness of remote, group-based trauma treatments is growing. Frueh et al. (2007) found no differences in PTSD symptom outcome between videoconferencing and in-person group CBT, though in-person participants felt more comfortable and adhered better to homework. A pilot trial by Morland et al. (2011) also demonstrated the feasibility of videoconferencing for group cognitive processing therapy, with similar outcomes in symptom improvements and adherence. These results were supported in a larger RCT (Morland et al., 2014). However, these studies primarily involved adult male military participants. Individual TF-CBT via videoconferencing has been shown to improve PTSD symptoms among CYP (Stewart et al., 2017; Stewart et al., 2020). From a clinical perspective, group-based delivery provides an opportunity to be with others with similar experiences, which may reduce the stigma and isolation often experienced by people with PTSD (Barrera et al., 2013). A supportive group environment can also allow the rebuilding of safety and trust, which may be particularly important for people with chronic PTSD (Barrera et al., 2013). Yet, there are risks, including vicarious traumatisation through hearing the detailed trauma accounts of others, or unhelpful comparisons with others, in which group members either minimise or amplify their own trauma (Barrera et al., 2013). Moreover, in a group format, there can be limited time for each individual (Barrera et al., 2013). In TRT, these risks are reduced by leaders supporting young people in keeping their trauma narratives brief and not overly detailed. Consequently, the exposure dose in TRT remains low, which may offer protection but could affect outcomes. Looking to the wider literature, group-based teleconferenced therapies appear to be as good as in-person therapies for CYP with other diagnoses, such as eating disorders (Hamadi et al., 2021) and social anxiety (Peros et al., 2021).

Although there is a high need for mental health support among individuals with experience of forced migration, there are multiple barriers to accessing in-person interventions. Online delivery may offer a promising alternative for this population (Abasi et al., 2025), yet socio-economic and structural barriers, including access to technology, linguistic challenges, and privacy limitations, remain (Weith et al., 2023). Furthermore, these trauma experiences may be exacerbated by resettlement stress as refugee CYP are dealing with issues of discrimination, social isolation, acculturation and uncertain legal status during a developmentally critical time (Abdi et al., 2023; Fazel and Betancourt, 2018). Online-delivered TRT represents one approach to mitigating access barriers by enabling remote participation in trauma-focused care; participatory adaptations informed by refugee youth engagement were incorporated to support accessibility and engagement (Pérez-Aronsson et al., 2022), although structural constraints may continue to affect participation.

This study aimed to examine the feasibility and acceptability of online-delivered TRT for CYP in Sweden who had experienced forced migration and reported symptoms of PTSD. Feasibility was defined as the ability to deliver the intervention as intended in a real-world digital context, including platform functionality, management of technical issues, group formation, and delivery of core intervention components. Although guidance addressing such challenges was included in the intervention manual, it remains essential to evaluate these issues during actual implementation, because feasibility studies assess delivery under real-world conditions rather than ideal circumstances. The feasibility of outcome measurement was assessed through completion of pre- and post-intervention questionnaires. Acceptability was defined as participants' subjective experiences of, and satisfaction with, the intervention content, delivery modality, and facilitator relationships.

## 2 Methods

The study employed an open, single-arm trial design. A mixed-methods approach was used, incorporating both quantitative and qualitative data. Fidelity checklists were completed by the TRT group leaders to monitor adherence to both online delivery standards and the intervention model. Self-report questionnaires were used to assess changes in psychological symptoms from pre-intervention to post-intervention. A focus group discussion and individual interview were held to explore participants' perspectives. The research team also took field notes based on conversations and email exchanges with the TRT facilitators during the trial period. Data collection took place between December 2021 and August 2022.

### 2.1 Intervention

**Fig. 1:**
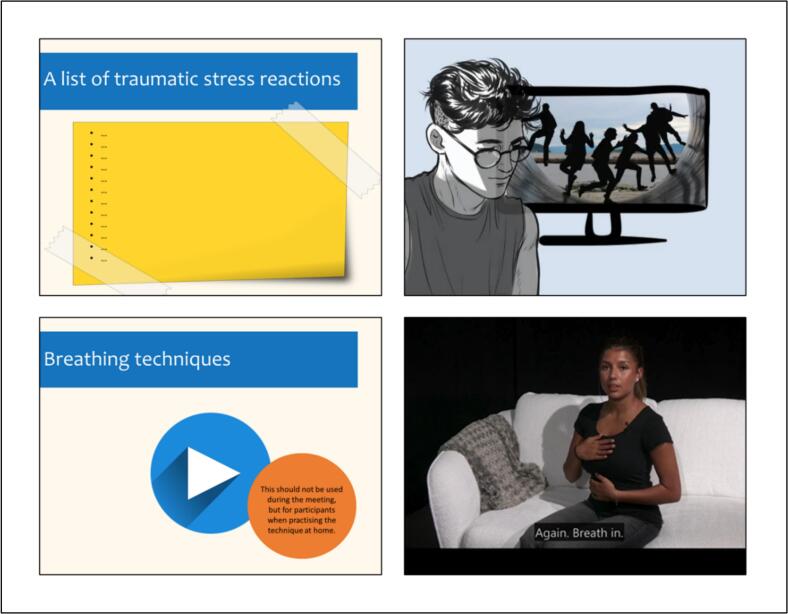
Example visual aids, which included editable slides where lists could be created, digital cartoons to introduce techniques, and links to video demonstrations for participants to use at home to practice techniques between sessions.

The original TRT manual comprises five sessions (90 min each), delivered on a weekly basis. It is delivered by professionals working with children (e.g., school counsellors, social workers) after a 3-day training. TRT is based on the core principles of Trauma-Focused Cognitive Behavioural Therapy (TF-CBT). It addresses key trauma symptoms through psychoeducation, relaxation and visualisation techniques, cognitive restructuring, exposure, and future goal setting, while caregiver sessions support parents in reinforcing these skills at home and improving their own well-being. When TRT was adapted to the Swedish context, an introductory session and a closure session were added, resulting in seven sessions (Sarkadi et al., 2018). In the online adaptation process (Pérez-Aronsson et al., 2022), it was decided that the first session should be a one-to-one session between the participant and a group leader. Thereafter, there would be six group-based sessions. The content for the sessions is outlined in Table 1, and examples of visual aids are shown in Fig. 1. When adapting the TRT intervention for online delivery, several changes were made to ensure safety, promote participation, and support learning (Pérez-Aronsson et al., 2022). Safety measures included conducting the Cantril Ladder (Cantril, 1965; Mohammed and Warner, 2024) with participants before each session to understand their current state of life satisfaction, with group leaders prepared to implement suicide screening if low ratings were reported. In addition, a clear protocol for managing adverse reactions during sessions was developed. This involved the use of private locations, keeping cameras on, a shared stop signal, direct communication channels to group leaders, and a nominated adult for in-person support. To encourage participation, adaptations involved holding individual pre-group meetings, incorporating fun activities into the sessions, providing accessible information, co-scheduling sessions with participants, sending reminders, and offering starter kits. Learning was supported by keeping sessions short (60–90 min) with regular breaks, maintaining small group sizes (4–7 people), being mindful of camera positioning, using visual aids (see Fig. 1), and favouring live, interactive demonstrations over pre-recorded content.

**Table 1:** Overview of intervention content.

Session	Format	Content
1	Individual	• Short presentation of the group leaders • Brief overview of TRT and the techniques that will be covered • Overview of common rules for the group • Technical support to enable participation with an active mic and video • Overview of safety protocol
2	Group	• Welcome, reminder of what TRT is and how group meetings will be conducted • Getting to know each other through games and activities • Agree and write down common rules for the group • Psychoeducation: Talk about how people react to being exposed to trauma • Normalise reactions to traumatic events • Make three lists together with the group: (i) Traumatic events; (ii) Traumatic stress reactions; and (iii) Things that remind you of the trauma • Practice the guided visualisation technique ‘Safe Inner Place’
3	Group	• Welcome, reminder of the purpose of the TRT groups • Recall the common rules for the group and follow up on assigned homework • Practice techniques for creating inner images: *Screen Technique; Hand and distancing techniques; Framing; Positive counter-images; Locking in the image; Helpers in the world of imagination; To switch off* • Practice bilateral stimulation • Working with nightmares • Introduce ‘Worry time’ - a technique for disturbing worries and thoughts • Other techniques, if relevant to the group's traumatic stress reactions: *Techniques related to hearing; Techniques related to odour; Techniques related to touch; Distraction methods*
4	Group	• Welcome, reminder of the purpose of the TRT groups • Recall the common rules of the group and follow up on assigned homework • Psychoeducation: Increased physiological response • Practice relaxation techniques • Practice breathing control • Practice positive self-talk • Practice using a fear thermometer • Provide sleep advice • Provide information on activity planning
5	Group	• Welcome, reminder of the purpose of the TRT groups • Recall the common rules of the group and follow up on assigned homework • Psychoeducation: Avoiding things that remind you of the trauma • Participants each make a list of reminders • Participants make a personal fear ladder • Plan for real-life exposure
6	Group	• Welcome, reminder of the purpose of the TRT groups • Recall the common rules of the group and follow up on assigned homework • Exposure to a traumatic memory: drawing, writing or talking • Planning for the future
7	Group	• Overview of the techniques covered across all the group meetings • Positive feedback to each participant • Link participants to others in their network or local area for support and follow-up • Diploma and celebration

In the present study, the sessions were facilitated by a Swedish-speaking, female school counsellor and a Swedish- and Dari-speaking, male NGO worker. Both had attended the 3-day TRT training course, and the NGO worker had previously delivered several TRT groups. Professionally trained interpreters were hired by the school where the participants were recruited. The TRT manual was shared with them ahead of TRT group commencement, so they could familiarise themselves with intervention content and language. Dari interpretation was carried out by the Dari-speaking facilitator.

No caregiver sessions were delivered as part of this trial.

### 2.2 Participants

Participants were eligible for the study if they (1) were aged 15 years or older, (2) had experienced forced migration, (3) scored above the threshold (≥17) for PTSD on the 8-item Children's Impact of Event Scale (CRIES-8) (Perrin et al., 2005), (4) had access to the internet, and (5) were able to use a device to access the online TRT sessions. Participants were recruited via an upper secondary school in the South West of Sweden. No upper age limit was applied, as students in this setting may be older due to interrupted or delayed education following forced migration. No additional exclusion criteria were applied to maximise accessibility. The school counsellor provided study information sheets and conducted the CRIES-8 with interested students. Those who scored above the threshold proceeded to complete an online informed consent form. In addition to the outcome measures detailed below, participants were asked to provide basic demographic information, including their age, gender, and current use of prescribed medications.

### 2.3 Measurements

#### 2.3.1 Fidelity checklists

To assess the consistency of intervention delivery, group leaders completed checklists following each session to capture key indicators of intervention fidelity. These included the date, session length, whether the Cantril Ladder was administered, for how many people additional safety measures were required, questions regarding digital delivery (private room, cameras on, individual contact with participant e.g., via chat function, intervention elements amended due to digital format, technical issues, digital platform used), and a structured list of predefined session elements. This process supported systematic monitoring of adherence to both online delivery standards and the intervention model.

#### 2.3.2 Questionnaires

The participants were asked to complete questionnaires regarding symptoms of PTSD, depression and anxiety, as well as life satisfaction. These were administered around a week before the first session and at the end of the final session. The questionnaires were available in Swedish, English, Arabic, Dari, Farsi, Somali and Tigrinya. Where possible, validated translations were used. However, when necessary (e.g., for the Cantil Ladder and for Tigrinya translations), the native language skills of the research group were used to produce translations.

##### 2.3.2.1 Children's Revised Impact of Events Scale (CRIES-13/CRIES-8)

The CRIES-13 (Perrin et al., 2005) consists of 13 items that assess PTSD symptoms. Each item is evaluated based on how frequently it has occurred in the past week (None = 0, Rarely = 1, Sometimes = 3, and A lot = 5). The questionnaire contains four intrusion items, four avoidance items, and five arousal items. The total scores for the scale range from 0 to 65, with a cut-off score of 30 or higher. The instrument has a high test-retest reliability correlation (*r* = 0.85) (Perrin et al., 2005), and the overall score has demonstrated strong internal consistency and successfully identifies over 75% of children with or without a PTSD diagnosis (Verlinden et al., 2014). The CRIES-8 includes only the intrusion and avoidance items. In the present study, the CRIES-8 was used to screen the CYP (eligibility cut-off of ≥17) as its brevity made it feasible for initial recruitment in a school setting, and the CRIES-13 was used as an outcome measure because it captures a broader range of PTSD symptoms, making it more suitable for measuring change over time.

##### 2.3.2.2 Patient Health Questionnaire (PHQ-9)

The PHQ-9 (Kroenke et al., 2001) consists of nine items designed to screen, diagnose, monitor, and assess the severity of depression. Each item is evaluated based on how often it occurred in the past two weeks (Not at all = 0, Several days = 1, More than half the days = 2, Nearly every day = 3). The total score on this scale ranges from 0 to 27, with established cut-off scores of 5, 10, 15, and 20, indicating mild, moderate, moderately severe, and severe symptoms, respectively. This instrument has demonstrated high internal consistency (Cronbach's α = 0.86 and 0.89 in two primary care samples) and a high test–retest reliability correlation (*r* = 0.84) (Kroenke et al., 2001). Construct validity and diagnostic validity for major depression have been confirmed in multiple studies, with sufficient sensitivity (0.71 to 0.87) and high specificity (0.88 to 0.95) identified for scores of PHQ-9 ≥ 10 (Gilbody et al., 2007). The PHQ-9 has also proven effective in evaluating treatment outcomes, and a change of 5 points is suggested to indicate a clinically significant change (Löwe et al., 2004). It has been tested with adolescents (Richardson et al., 2010) and was utilised as a self-report measure in the current study.

##### 2.3.2.3 Generalised Anxiety Disorder-7 (GAD-7)

The GAD-7 (Spitzer et al., 2006) was initially created to identify generalised anxiety disorder. However, it has also been commonly utilised to evaluate the severity of more general anxiety symptoms. Each item is assessed based on the frequency of its occurrence over the last two weeks (Not at all = 0, Several days = 1, More than half the days = 2, Nearly every day = 3). The total scores can vary from 0 to 21, with cut-off scores of 5, 10, and 15 indicating mild, moderate, and severe symptoms, respectively. It has demonstrated high internal consistency (Cronbach's α = 0.92), high test-retest reliability (*r* = 0.83), and appears to perform adequately as a marker of symptom severity (Spitzer et al., 2006). The GAD-7 has been tested with adolescents (Löwe et al., 2008) and was used as a self-report measure in the current study.

##### 2.3.2.4 The Cantril Ladder

The Cantril Ladder is a widely used single-item measure of life satisfaction. Respondents are asked to place themselves on a ladder from 0 (worst possible life) to 10 (best possible life), based on their current life circumstances (Cantril, 1965). The scale has been extensively applied in large-scale surveys and demonstrates moderate test–retest reliability (*r* = 0.6–0.7) (Helliwell et al., 2020). It has been tested with refugee youth reporting symptoms of PTSD, and showed moderate concurrent validity with validated measures of depression and self-efficacy (Mohammed and Warner, 2024).

#### 2.3.3 Qualitative data

Both the focus group discussion and individual interview were held online via a secure video conferencing platform, facilitated by a member of the research team experienced in qualitative interviewing and group facilitation (SGL). The aim was to elicit participants' perspectives on the acceptability, relevance, and perceived impact of online-delivered TRT. A semi-structured topic guide was used to ensure consistency across discussions while allowing flexibility to follow relevant topics introduced by participants (see Table 2). The focus group lasted 42 min, and the individual interview 35 min. Both were audio-recorded and transcribed verbatim.

**Table 2:** Question guide.

Question	Prompts
Tell me about when you first heard about TRT	*How come you wanted to take part?*
What do you think about what you did in the group?	*Is there anything you learned in TRT that you kept on using after the sessions?*
	*Would you have liked more/less of something?*
If you had a friend with trauma, what would you teach them?	
Tell me about how you felt in the group.	*How was it with the others in the group?*
	*Describe the engagement in the group. Talkative?*
	*What did it feel like to hear others tell their stories?*
	*Do you still see any of them?*
Describe your relationship with the group leaders.	*What was your experience of the ‘get to know the leader’ session?*
If you were to tell a friend about the group, what would you say?	*Would you recommend the group to a friend?*
What was it like to have the sessions online?	*Any advantages?*
	*Any disadvantages?*
	*Where did you attend the sessions?*
	*Privacy at home?*
	*Technical issues?*
	*Used to using the platform?*
	*Using the camera?*
What do you think the difference would have been to have TRT face-to-face?	
How would you prefer TRT to be delivered?	

### 2.4 Analysis

Descriptive statistics were calculated to summarise participant characteristics, intervention attendance, session content fidelity, completion rates, and psychological symptomatology.

The focus group and individual interview transcripts were analysed using deductive content analysis (Elo and Kyngäs, 2008). Individual participant utterances were used as the unit of analysis. Three predefined categories were developed based on the research question: (i) feasibility; (ii) acceptability, and (iii) perceived effectiveness. Each category was defined and organised into a codebook to guide the coding process and promote consistency and rigour. The primary coding was conducted by EGS. SGL and GW served as co-analysts; while they did not directly code the material, they reviewed the coded data, provided feedback, and participated in discussions to ensure the accuracy, clarity, and interpretive validity of the coding decisions. This consensus-based review process helped to enhance the credibility and trustworthiness of the analysis.

Joint display was used to facilitate the integration of quantitative and qualitative findings (Guetterman et al., 2015). This enabled the comparison and triangulation of outcome trends with participant-reported experiences, supporting a more comprehensive interpretation of the feasibility and acceptability of the intervention.

### 2.5 Ethical considerations

The study was approved by the Swedish Ethical Review Authority on 4th June 2021 (Ref. 2021–02160). All participants were aged 15 years or older and thus could self-consent according to Swedish ethics law. A comprehensive safety protocol was implemented. Prior to each session, participants completed a brief life satisfaction check using the Cantril Ladder (Mohammed and Warner, 2024). A score below 4 triggered a structured screening for suicidal ideation, conducted by the group leaders. Additionally, one group leader monitored participants during sessions and was available to initiate direct contact if concerns arose. For each participant, a trusted adult was identified and informed of the session schedule and the participant's location. This adult could be contacted if immediate in-person support was required. While these safeguards were in place, they were not required during the study.

## 3 Results

### 3.1 Sample

A total of 16 participants were enrolled in the study. The sociodemographic characteristics of the sample are presented in Table 3.

**Table 3:** Demographics of sample.

Age (years)	19.7 (SD = 1.85)
Gender (% male)	62.5% (*n* = 10)
Country of birth	Afghanistan (n = 9), Syria (*n* = 2), Lebanon (n = 2), Ethiopia (*n* = 1), Iran (n = 1), and Uganda (n = 1)
Years of school	9.0 (SD = 4.37)
Years in Sweden	4.5 (SD = 1.90)
Accommodation	Family home (n = 3), Residential care home (n = 1), Own accommodation (*n* = 4), Rents room (*n* = 5), Student accommodation (n = 1), Hotel (n = 1), No accommodation (n = 1)
Residence permit	Permanent (n = 1), Temporary (*n* = 8), Waiting (n = 3), Rejected (*n* = 3), Don't know (n = 1)
Current use of prescribed medication	25.0% (n = 4)

### 3.2 Feasibility

#### 3.2.1 Platform functionality and management of technical issues

The TRT sessions were delivered via Google Meet, as this was the platform routinely used by the recruitment school. Google Meet does not provide a private chat function; therefore, administration of the Cantril Ladder at the beginning of each session was conducted via SMS rather than through the teleconferencing system's private chat feature, as outlined in the intervention manual.

While the majority of participants attended with their cameras on, most did not situate themselves in a private room. This was echoed in the interview and focus group, with the young people explaining that having their cameras on made it feel like they were there in person, whilst also benefiting from the safety of their own home. Some of them expressed that they were able to have privacy in their own rooms, whilst others shared that family members disturbed them at times.

Several technical issues were reported. Field notes indicated that one participant experienced repeated disruptions due to unstable internet connectivity. The interpreter also encountered connection issues, which resulted in their video freezing intermittently. Another participant faced difficulties with their camera. During one session, a participant observed that the images of the group leaders had disappeared. However, this issue was related to using a mobile phone; the leaders were able to see each other without any interruptions throughout the session. Young people from the focus group also expressed that some tasks were challenging to carry out online, for example, drawing and sharing pictures. The group leader resorted to sharing these via a link after the session.

*“For example, when we had a meeting about drawing, we couldn't see what he [the leader] had drawn”*

*Participant 3, Female, Focus group*

#### 3.2.2 Group formation and delivery of core intervention components

Three groups were formed based on the languages spoken by participants to avoid having interpreters simultaneously. Group size ranged from four to six. However, correspondence with the TRT facilitators informed that two of the groups, which were being delivered in parallel, merged after session three, as two participants dropped out of one group, leaving only two participants. The decision to merge was taken together with the participants.

The session dates on the fidelity checklists and correspondence with the TRT facilitators informed that, instead of delivering the sessions weekly, they were held twice a week, allowing all sessions to be completed within four weeks. Within the focus group, it emerged that the group setting could lead to challenges due to certain participants arriving late, causing delays for the rest of the participants. Several participants proposed directly to the facilitators that the session duration be limited to a maximum of an hour. In response to this, the facilitators informed that the sessions were reduced from 90 to 60 min. In the focus group, the young people expressed how, even with these shorter sessions, videoconferencing was tiring, resulted in minimal movement and strained their eyes.

*“You become tired when you are home because you are staring at a screen the entire time, and it makes your eyes weak.” Participant 2, Female, Focus group.*

Fidelity checklist data indicated that the group leaders delivered all of the predefined session elements as intended, except for the final session. During this session, the review of techniques took place, as did giving positive feedback. However, the group leaders did not link the participants to others for support, and the ‘diploma and celebration’ element did not take place. No safety protocol measures, beyond routine administration of the Cantril Ladder, were required throughout the trial.

#### 3.2.3 Outcome measurement

Of the 16 participants, nine completed the post-intervention survey, three took part in a focus group discussion (1 male, 2 female), and one (male) took part in an individual interview. The pre-intervention characteristics of completers (*n* = 9) and non-completers (*n* = 7) are presented in Table 4. The descriptive statistics indicate that attrition may have introduced some bias, with the youth completing the study seemingly less likely to be female and reporting a higher level of psychological symptoms. The mean scores on the CRIES-13, PHQ-9, GAD-7 and Cantril Ladder pre- and post-intervention are presented, together with standard deviations, in Table 5.

**Table 4:** Pre-intervention characteristics for all participants versus completers.

	Completers (n = 9)	Non-completers (n = 7)
Age	19.6 (SD = 1.94)	19.9 (SD = 1.86)
Gender (% male)	77.8%	42.9%
CRIES-13	45.2 (SD = 14.8)	38.6 (SD = 15.00)
PHQ-9	18.1 (SD = 7.32)	13.4 (SD = 5.94)
GAD-7	15.1 (SD = 6.05)	10.7 (SD = 3.77)
Cantril Ladder	7.44 (SD = 2.35)	6.57 (SD = 2.15)

**Table 5:** Pre-post mean scores and standard deviations for the CRIES-13, PHQ-9, GAD-7 and Cantril Ladder.

	Pre-intervention (n = 9)	Post-intervention (*n* = 9)
CRIES-13	45.2 (SD = 14.8)	41.6 (SD = 9.41)
PHQ-9	18.1 (SD = 7.32)	16.6 (SD = 6.82)
GAD-7	15.1 (SD = 6.05)	13.3 (SD = 4.09)
Cantril Ladder	7.44 (SD = 2.35)	6.22 (SD = 2.77)

### 3.3 Acceptability

#### 3.3.1 Intervention content

Within the focus group and interview, it emerged that the young people were motivated to take part in the intervention in the hope that it would alleviate trauma symptoms. Some felt that it did reduce symptoms, including sleep problems and panic attacks, and referred to specific techniques they felt had helped. A few talked about how the intervention had reduced stress and improved their health in general.

*“Yes, so [the leader] helped me with this [panic attacks], before it would happen to me in school, it was really hard, I just wanted to hit something, you know, I was really stressed, then she [the leader] told me just breathe, then at the toilet and close the door and start breathing.” Participant 4, Male, Interview.*

However, all raised how the techniques and the intervention as a whole only provided momentary relief from their trauma symptoms, with the underlying and ongoing stressors not addressed. They described how the distress of, for example, whether they would be granted a residency permit by the migration agency, or the fear of family members in war-torn areas being killed, remained. Whilst one young person expressed that the intervention did not help at all, most of the young people felt that it was still helpful to manage symptoms, whilst acknowledging that the underlying cause remained.

*“For example, in a land where there is war, okay, we can be okay for a while, but when we are told every day that someone has died, and another person dies, thousands of people, so this group doesn't help someone who has stress” Participant 3, Female, Focus group.*

Whilst it seemed that some techniques were used in everyday life post-intervention, some young people appeared to stop using them once they felt that their symptoms had improved.

#### 3.3.2 Delivery modality

The acceptability of the 1:1 introductory session varied across the young people. Most found it helpful to gain insight and a sense of what to expect ahead of the intervention, whilst others found that it didn't add anything beyond what they already knew and that the starter session and starter package were not needed.

*“It's okay if we don't have it [starter package]” Participant 2, Female, Focus group.*

Whereas others shared that beyond the information provided, the introductory session contributed to building a sense of trust, safety and future engagement in group sessions. One young person expressed how they preferred the 1:1 session over the group setting. They found that it helped them open up about their experiences, which they did not feel able to do in a group.

*“I think it is easier to talk to the leaders first, and then it becomes easier because you want to take part in the group and you feel, as in it feels secure…yes and not so strange if you know the leader a little bit beforehand.” Participant 1, Male, Focus group.*

Whilst there were some perceived benefits of being online, such as being in the comfort of one's own home, most felt it would have been better in person. They found that certain tasks, such as watching digital cartoons, were not particularly helpful, as they found it difficult to concentrate. They also explained that meeting in person would make the intervention more sociable and facilitate better relationship building between participants, contributing to it feeling safer to share thoughts and experiences. This seemed particularly important in cases where participants did not know each other beforehand, as some articulated how it is difficult to share feelings with people you don't know.

*“I thought that if there is anyone I don't know, it is hard to talk just how you feel” Participant 2, Female, Focus group.*

However, one young person explained how they would have felt more comfortable in sharing their thoughts and feelings with group members they did not know. This appeared partly due to being shy but also rooted in feeling stigmatised.

“*You know they [the leader] said that they said to me that you can talk, you know, I know I can talk, but I never want anyone from my school, or my friends knowing about what happened, I just listen.”*

*Participant 4, Male, Interview*

#### 3.3.3 Facilitator relationships

All young people in the focus group and interview spoke about the importance of the leaders' qualities in relation to the acceptability of the intervention. They explained how they were kind, engaging, maintained participants' focus, helped participants feel heard and understood, enabling young people to open up about their experiences. A few raised how this was key to their continued involvement and engagement in the group.

*“Yes, if anyone doesn't know me, talks to me, anything that I can't accept, I will not come to the group, but they [the leader] were really kind”. Participant 4, Male, Interview.*

Some also discussed how there was an easy and clear line of communication with the group leader if they could not attend or were late. However, one young person found the communication stressful, receiving multiple missed calls when they were late.

## 4 Discussion

### 4.1 Feasibility

The lack of private spaces and connections during sessions may have impacted the confidentiality and comfort levels of participants. This was anticipated as an issue in the adaptation phase (Pérez-Aronsson et al., 2022) and has been noted as a procedural issue in telepsychiatry research and program development (Frueh et al., 2000). Connecting to videoconferencing platforms from public locations poses immediate security risks, such as unsecured networks or eavesdropping. Even in secure settings, a single screenshot or shared image can compromise confidentiality by enabling identity tracing and social network reconstruction (Kagan et al., 2024). Moreover, individuals who have experienced forced displacement are disproportionately affected by privacy concerns compared to non-displaced clients (Weith et al., 2023). This underscores not only the importance of securing private settings for virtual therapeutic interventions but also clear privacy rules among participants.

Technical issues, though relatively infrequent, posed barriers to participation and highlighted the vulnerability of online delivery to connectivity-related disruptions. This challenge was anticipated as it was raised in the needs assessment phase of the adaptation process (Pérez-Aronsson et al., 2022). The proposed solution was to address the practicalities of meeting online in the first one-to-one meeting (Pérez-Aronsson et al., 2022). While the group leaders in the present study confirmed that the one-to-one meetings took place, it is not clear how much time was given to the topic of technical practicalities and the issue regarding seeing multiple attendees on a mobile phone screen would not have surfaced with only two people attending. One notable challenge is the continual evolution of technology. Keeping abreast of technological innovations and market conditions that support teleconferencing has been raised as a procedural issue in telepsychiatry research and program development (Frueh et al., 2000).

After two participants dropped out, two groups were combined. The facilitators adhered to the online TRT manual's recommendation of four to seven participants per group to maintain therapeutic dynamics. However, merging the groups after the intervention had already started may have impacted group cohesion. The extant literature shows that group cohesion is an important factor in group therapy (Burlingame et al., 2018). Group size plays a role; meta-analysis showed that group size moderated the relationship between cohesion and participant outcomes, but not when the group consisted of more than nine members (Burlingame et al., 2018).

Delivering sessions twice weekly instead of once per week enabled the intervention to be completed within a four-week timeframe. This compressed timeline may have supported participant engagement by maintaining continuity and momentum between sessions. However, the accelerated pace may have affected the consolidation of learning. The length of each session was also reduced from 1.5 h to 1 h. The adaptation was made at the request of participants to improve focus and comfort, yet they still indicated that this amount of time videoconferencing could be tiring. It aligns with the input from the youth co-researchers during the development of the online TRT manual (Pérez-Aronsson et al., 2022) and the concept of ‘videoconference fatigue’ in which prolonged use of a videoconference platform can result in feeling overwhelmed, frustrated or exhausted (Döring et al., 2022).

Fidelity checklist data indicated that there was a high level of adherence to the intervention content; even though sessions were shortened to one hour, the TRT group leaders covered the predefined elements for each session. The only exception was the final session, in which there was no ‘diploma and celebration’ element, and no structured link to external support networks. Shapiro and Ginzberg (2002) emphasise the potential of termination rituals to consolidate therapeutic gains, rework past separations, and foster a stronger sense of self. They can offer symbolic closure and a structured way to process the emotional complexity of endings. In the context of group-based trauma support for CYP, omitting a planned termination ritual like a diploma ceremony may inadvertently bypass important emotional work. Shapiro and Ginzberg (2002) argue that rituals help internalise progress and allow for recognition, mutual acknowledgement, and grieving. Without such closure, the group's ending may replicate feelings of abrupt loss or invisibility. Thus, the decision to skip the celebration should be revisited with care.

### 4.2 Acceptability

The young people taking part in the focus group and interview voiced that having the intervention in person would contribute to better relationship building. This was despite the participatory adaptations made to TRT to try to foster participation, such as the integration of games and limiting the group size to enable interaction (Pérez-Aronsson et al., 2022). As such, online delivery may also have impacted group cohesion. This corroborates findings from a study in which those in an online group felt less connected to each other, scoring lower on a group cohesion scale compared to the in-person group (Lopez et al., 2020). The participants also reported concerns related to psychological safety and willingness to share, reflecting broader trust-related challenges that have been widely observed in the context of trauma experience and forced migration (Essex et al., 2022).

Participants reported difficulty maintaining attention with visual aids to the TRT group leaders, suggesting that alternative engagement tools may be needed in remote delivery contexts. This aligns with research on visual aids in video lectures, which found that students exposed to fewer visuals reported a higher effectiveness of examples in illustrating concepts and a better overall learning experience (Fish et al., 2016).

The young people taking part in the focus group and interview perceived certain symptoms to have improved, whilst acknowledging that the underlying stressors remained. Although quantitative measures of adverse events were not collected, qualitative findings indicate recurrent experiences of bereavement, particularly the loss of loved ones in participants' countries of origin. Furthermore, group leaders noted that the political situation in Afghanistan was particularly unstable during the intervention period, from which a large proportion of participants originated. Some participants were approaching the age of 18, which may be a critical threshold for forcefully displaced youth due to loss of child-specific protection related to legal status and housing. This is consistent with prior research highlighting the distinct psychosocial stressors faced by forcibly displaced youth (Fazel et al., 2012).

### 4.3 Strengths and limitations

The open, single-arm trial format allowed for the delivery and evaluation of online TRT in a naturalistic setting, closely reflecting real-world conditions. The use of a mixed-methods approach provided a comprehensive evaluation of the intervention, enabling a richer understanding of the intervention's feasibility and acceptability.

However, several limitations must be acknowledged. Participant screening relied on the CRIES-8, which focuses on intrusion and avoidance symptoms and may not identify individuals with PTSD who primarily exhibit hyperarousal or other symptom clusters (Perrin et al., 2005). This limitation is particularly pertinent in culturally diverse samples, as avoidance symptoms may manifest differently across cultures, potentially affecting diagnostic accuracy and treatment outcomes (Hinton and Lewis-Fernández, 2011). Future research could consider structured clinical interviews to assess a wider range of PTSD symptoms, trauma severity, and ongoing contextual stressors. Yet, it's important to remember that TRT is non-specialist care, so clinical interviews wouldn't be typical in the recruitment process.

Online delivery of TRT offers the advantage of recruiting participants from diverse geographic locations, which may help reduce barriers related to social stigma and facilitate help-seeking compared with traditional in-person services (Knappe et al., 2019). However, in this pilot study, recruitment was limited to a single school, which may have reduced perceived anonymity and affected engagement or group cohesion, even though confidentiality and privacy were emphasised as core group rules.

The small sample size and absence of a control group mean we are unable to draw causal inferences about the efficacy of the intervention. Detailed information on trauma type, socioeconomic background, and prior social or psychological support was not collected in this pilot study to minimise participant burden and align with the feasibility focus. Future research should include these variables to better contextualise findings and examine potential moderators of intervention outcomes.

It appears as though study dropout introduced some bias; while the completers and non-completers were similar in age, the completers seemed to include fewer females and a higher level of symptom burden. Similarly, there may have been selection bias in the qualitative findings, as very few participants chose to take part. The individual interview was conducted due to the emotional vulnerability of the participant, and was included to ensure that their perspective was not excluded. Although data may differ across interview modalities, flexibility in data collection is consistent with ethical qualitative research practice, particularly when discussing sensitive topics where participants may prefer a private setting (e.g., Baillie, 2019). The focus group included three participants; although smaller than typical recommendations, small groups can still generate meaningful exploratory data on shared experiences and group dynamics, while limiting transferability (e.g., Baillie, 2019).

Although the intervention was deliverable and completed with maintained fidelity, several context-related barriers (e.g., lack of private rooms and connectivity problems) affected group cohesion and task completion. These barriers suggest that while online TRT is feasible and generally acceptable, adaptations are needed to strengthen engagement and reduce implementation challenges in future trials. The omission of caregiver sessions may have reduced the intervention's potential effectiveness, and future research should examine outcomes when caregiver involvement is included.

### 4.4 Implications for practice

TRT group leaders should ensure that participants are adequately prepared for the technical demands of virtual sessions. If the videoconferencing platform allows, the leaders could be ‘pinned’ so that shifting views do not affect the visibility of the leaders throughout the session. Participants should be encouraged to arrange a quiet, uninterrupted environment with a secure internet connection for sessions; caregivers or schools may play a supportive role in facilitating this where needed. Not taking photos or screenshots during the sessions should also be added to the online TRT group rules in the manual. Despite a reduction in session duration, young people voiced screen fatigue, yet further shortening sessions may challenge the therapeutic dosage integrity, as adequate time is needed to rehearse and internalise strategies. Optimising session structure with micro-breaks and more interactive elements could be a possible solution. Fewer digital visual aids should be utilised. Elements that mark the completion of the program, such as the ‘diploma and celebration’, should not be overlooked.

### 4.5 Implications for future research

The study limitations underscore the need for larger, methodologically rigorous studies incorporating control groups to more robustly evaluate the impact of online TRT and improve the generalisability of findings. Future research could also examine whether specific aspects of the online delivery format, such as engagement strategies, group dynamics, or technological accessibility, may influence outcomes. Additionally, extended follow-up assessments could help identify any sustained or delayed effects that may not be captured in short-term evaluations.

## 5 Conclusions

The findings indicate that while online delivery was feasible, safe and generally acceptable, several implementation challenges emerged, including technical difficulties, privacy concerns, and limitations in group composition and closure procedures. Participants expressed a preference for in-person delivery, citing greater psychological safety and group cohesion. Despite reducing session duration, participants continued to report ‘screen fatigue,’ highlighting a tension between minimising online exposure and preserving sufficient time for practising and integrating therapeutic tools. Although the intervention was perceived as beneficial, it did not adequately address underlying systemic stressors. Future adaptations should prioritise strategies to enhance engagement, safeguard privacy, ensure meaningful closure, and integrate approaches that address systemic stressors. Further research is warranted to explore the potential efficacy of online-delivered TRT, as well as how technology access and engagement strategies influence outcomes. Longer-term follow-ups should be conducted to capture sustained or delayed effects beyond short-term evaluation.

## CRediT authorship contribution statement

Conceptualisation: GW & SGL; Quantitative data collection: SGL; Quantitative data analysis: GW; Qualitative data collection: SGL; Qualitative data analysis: EGS, SGL & GW; Manuscript writing: GW, SGL & EGS.

## Funding

This research was funded by the Kavli Trust (Grant ID A-321629). The funder has had no involvement in the design of the study, the collection, analysis or interpretation of data or in the writing of the manuscript.

## Declaration of competing interest

The authors declare that they have no known competing financial interests or personal relationships that could have appeared to influence the work reported in this paper.
